# Submandibular Gland Invasion From a Metastatic Lymph Node in Patients With Buccal Mucosal Squamous Cell Carcinoma: A Case Report

**DOI:** 10.1155/2024/7940535

**Published:** 2024-10-15

**Authors:** Moeka Bukawa, Kenji Yamagata, Satoshi Fukuzawa, Shohei Takaoka, Fumihiko Uchida, Naomi Ishibashi-Kanno, Hiroki Bukawa

**Affiliations:** ^1^Department of Oral and Maxillofacial Surgery, University of Tsukuba Hospital, 2-1-1 Amakubo, Tsukuba, Ibaraki 305-8576, Japan; ^2^Department of Oral and Maxillofacial Surgery, Institute of Medicine, University of Tsukuba, Tsukuba, Ibaraki 305-8575, Japan

**Keywords:** buccal mucosal squamous cell carcinoma, direct invasion, submandibular gland involvement, submandibular lymph node

## Abstract

This report presents a rare case of direct invasion from a metastatic submandibular lymph node (SMLN) to submandibular gland (SMG) in a resected specimen of neck dissection (ND) of buccal mucosal squamous cell carcinoma (SCC). The patient was an 82-year-old woman with a clinical diagnosis of the left buccal mucosal SCC (cT4bN2bM0, Stage IVB). The tracheostomy, modified radical neck dissection, buccal mucosal cancer resection including maxillary partial resection, mandibular segmentectomy, and reconstructive surgery with a plate and a free rectus abdominis flap were performed. Pathologically, the infiltrating SCC was observed in the SMG continuous with SMLN metastasis (pT4bN3bM0). No adjuvant therapy was performed for old age and oral intake dysfunction. Contrast CT detected the multiple lung and left scapula metastases at postoperative 5 months, which made the policy of best supportive care. Finally, she died 9 months after the surgery from distant metastases. SMG involvement from direct invasion from a metastatic SMLN is relatively rare. In our case, although the patient died from distant metastases, locoregional control was achieved through curative resection of the primary tumor and ND performed as one block with reconstructive surgery.

## 1. Introduction

The prevalence of histopathological submandibular gland (SMG) involvement removed after neck dissection (ND) for oral squamous cell carcinoma (OSCC) is generally low, ranging from 0% to 5% [1, 2]. Previous reports suggest that SMG may contribute to OSCC in four ways: (1) direct invasion from the primary carcinoma, (2) direct extranodal extension (ENE) from submandibular lymph nodes (SMLNs), (3) metastases to intraglandular lymph nodes (LNs), and (4) carcinoma growing along Wharton's duct [3]. Direct invasion of the SMG from the primary tumor is reported in 64.2% of cases, and from metastatic SMLNs in 23.9% of cases [2]. This report presents a rare case of direct invasion from a metastatic SMLN to the SMG in a resected specimen of a ND of buccal mucosal carcinoma, and the literature on the previous reports is also reviewed.

## 2. Case Report

An 82-year-old Japanese woman was referred to our hospital, complaining of swelling on the left side of her cheek. Her medical history revealed hypertension. She has a Kari flower-like swelling in the left buccal mucosa and was referred from a general dental clinic. Her personal history showed no history of alcohol consumption or smoking, and there was no family history of either.

**Figure 1:**
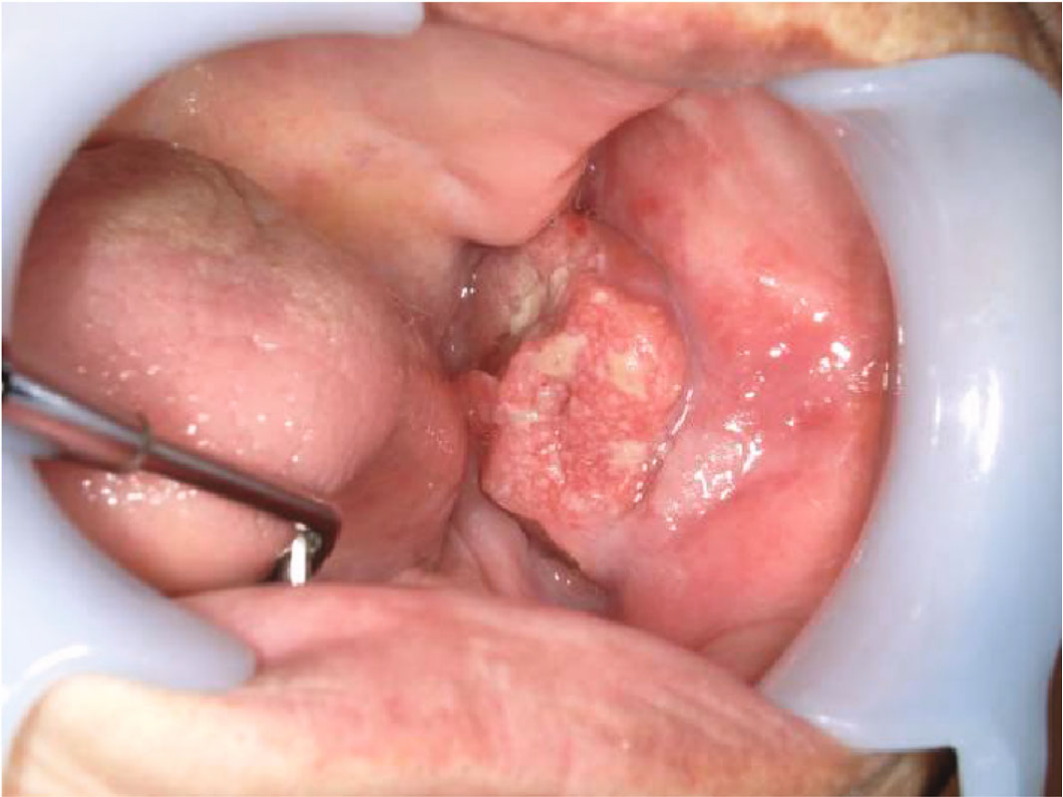
Intraoral finding. There is a hard, granular mass on the buccal mucosa. It measures 35 × 28 mm and has an ulcer in its center.

The patient's general examination revealed a performance status (PS) of 1, a height of 137.6 cm, and a weight of 49.9 kg with moderate build and good nutritional condition. Extraoral findings showed facial asymmetry with swelling and tenderness in the left buccal region and mandible. There was no inferior alveolar nerve palsy. Intraoral findings revealed a superficial granular mass measuring 35 × 28 mm with a central ulcer on the left buccal mucosa. The tumor was hemorrhagic and painful, with palpable surrounding induration (Figure 1). No salivary discharge was observed from the left Wharton's duct orifice.

**Figure 2:**
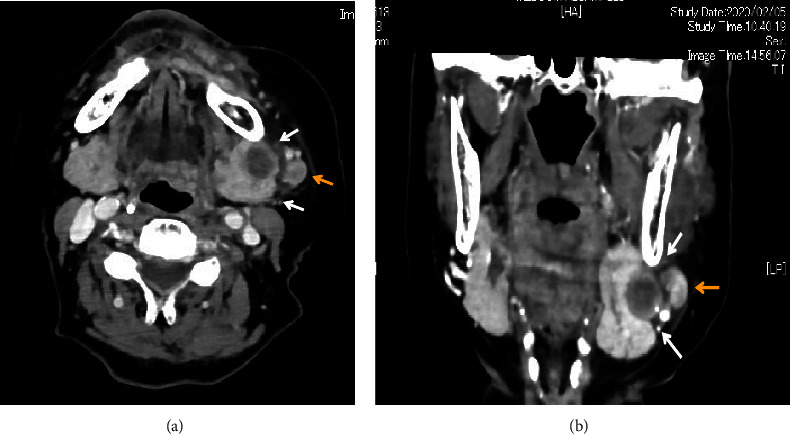
CT findings. (a) Axial view and (b) frontal view. A low-signal area of 11 mm in diameter was observed in the submandibular gland (white arrow). The submandibular lymph node was enlarged to 9.9 × 8.3 mm with internal necrosis (yellow arrow).

**Figure 3:**
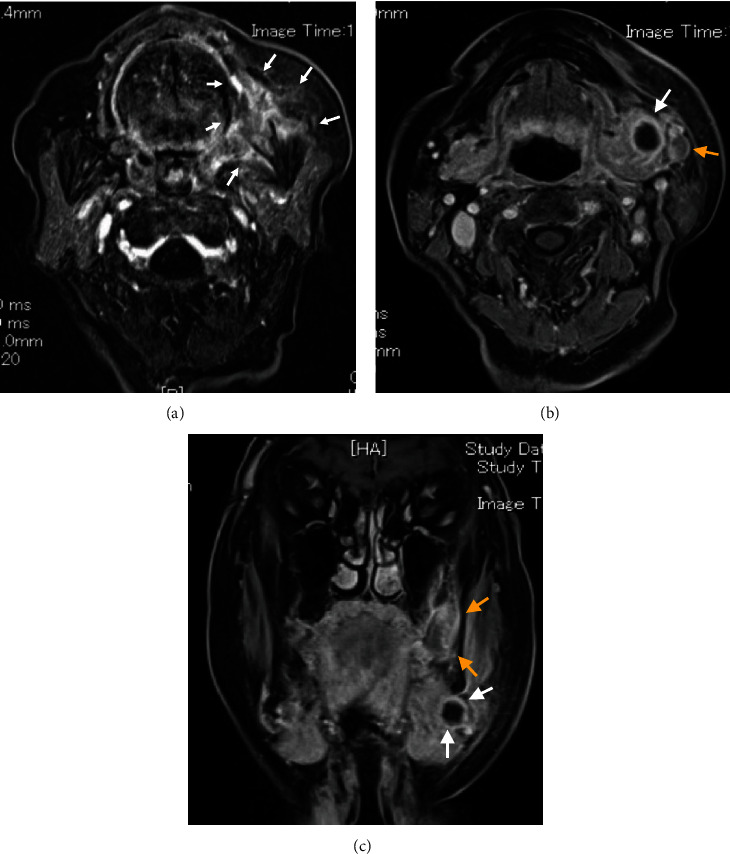
MR findings. (a) A 33 × 27 × 19 mm primary tumor is present in the buccal mucosa. The mass appears heterogeneous with high-signal intensity on T2-weighted image. The mass has indistinct borders and invades the masticatory muscle space and the mandible (axial position, T2-diffusion-weighted image). (b) Submandibular gland involvement (white arrow) and submandibular lymph node metastasis (yellow arrow). The submandibular gland is enlarged and low-signal areas of the metastatic submandibular lymph nodes are depicted (axial position, T2-weighted image). (c) There is no continuity between the submandibular gland involvement of submandibular lymph node metastasis (white arrow) and the primary tumor (yellow arrow) (frontal section, T2-weighted image).

**Figure 4:**
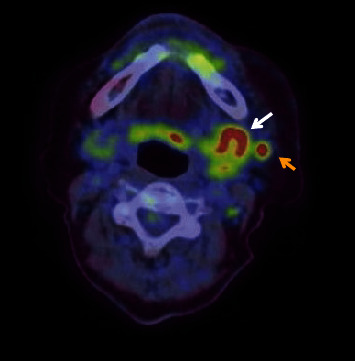
FDG-PET CT findings. FDG accumulation was observed in the submandibular lymph node (yellow arrow) (SUV max: 8.9) and in the submandibular gland (white arrow) (SUV max: 7.0).

Contrast-enhanced computed tomography (CT) revealed a low-signal area of 11 mm in diameter of the SMLN that had invaded the left SMG. Another left SMLN was enlarged to 9.9 × 8.3 mm and showed internal necrosis (Figures 2(a) and 2(b)). The magnetic resonance (MR) image depicted a 33 × 27 × 19 mm mass observed in the left buccal mucosa, which showed internal heterogeneity and high signal on T2-weighted image. The mass had indistinct borders and infiltrated the left-side maxillary bone, masticatory muscle space, and mandible (Figure 3(a)). Metastasis was observed in the left submandibular and superior internal deep cervical LNs. The left-side SMG was enlarged and showed a clear low-signal area, which was a metastatic SMLN not continuous with the primary tumor (Figures 3(b) and 3(c)). Fluorodeoxyglucose-positron emission tomography (FDG-PET) CT revealed FDG accumulation in the left buccal mucosal mass (SUV max: 16.4), left SMLN (SUV max: 8.9), and left SMG (SUV max: 7.0) (Figure 4). No lung or liver metastases were found. Laboratory results showed no abnormalities other than mild anemia (hemoglobin 10.7 g/dL). Based on these examinations, a clinical diagnosis of left-sided buccal mucosal carcinoma (T4bN3bM0, Stage IVB) was made.

A biopsy was performed on the left buccal mucosal mass under local anesthesia, and a histopathological diagnosis of well-differentiated squamous cell carcinoma (SCC) was made. The tracheostomy, modified radical ND (Levels I–V), buccal mucosal cancer resection, and reconstructive surgery with a plate and a free rectus abdominis flap were performed using a plate for reconstruction and a free rectus abdominis valve. The operation was performed in 11 h and 17 min (blood loss: 70 mL, blood transfusion: 280 mL). The mandibular bone was resected at the left second premolar and swung laterally. A 15-mm safety margin from the tumor was defined in the oral cavity, and the incision line was set posteriorly from the buccal mucosa to the palatal side of the maxillary first premolar, the lateral wall of the oropharynx anterior to the palatine tonsil, and the left side of the mandibular second premolar from the floor of the mouth. The primary tumor and the ND object were resected as one block.

The patient had good postoperative wound healing, but she was unable to take oral intake and declined percutaneous endoscopic gastrostomy (PEG) placement. Consequently, she was required prolonged eating and swallowing rehabilitation. A contrast-enhanced CT scan taken 1 month after surgery showed no signs of local recurrence, cervical LN metastasis, or lung metastasis. On the 41st postoperative day, the patient was transferred to another hospital for eating and swallowing rehabilitation. The patient was discharged from the hospital and became an outpatient after oral intake became possible. However, 5 months after surgery, contrast-enhanced CT revealed multiple lung metastases and left-sided scapular bone metastases, and the patient's age and wishes dictated best supportive care (BSC). Unfortunately, the patient's general condition worsened due to increased lung metastases, and she died 9 months after surgery, although there was no local or neck recurrence.

**Figure 5:**
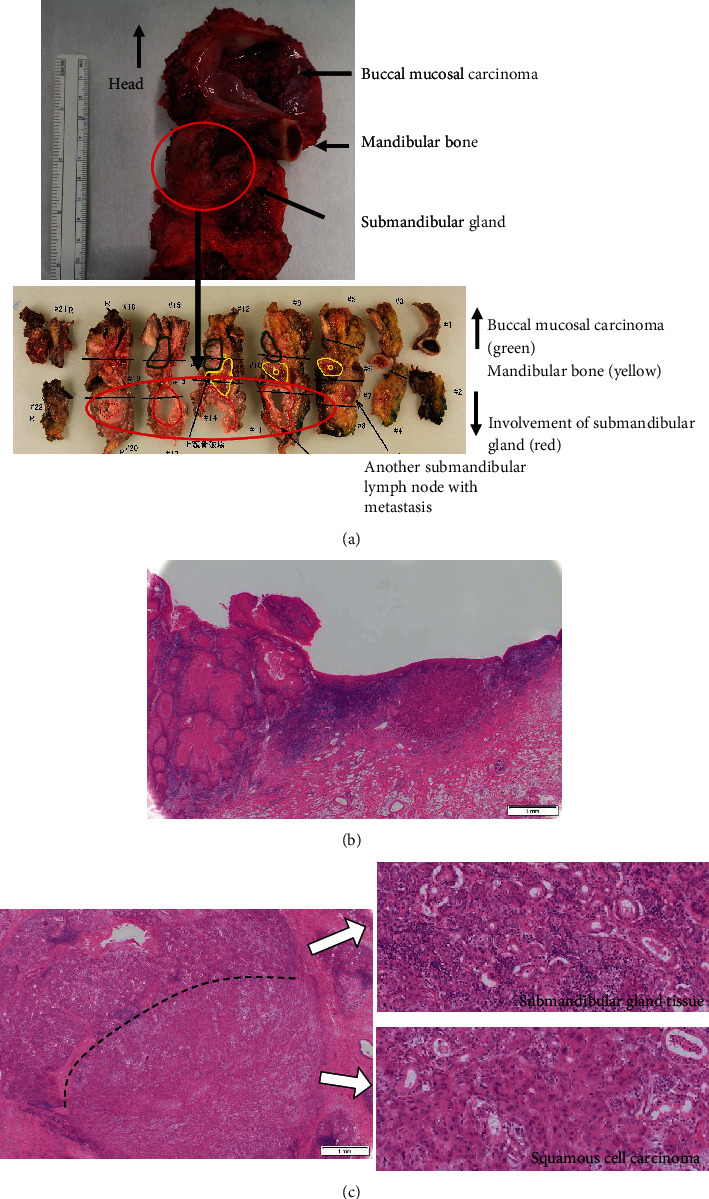
Histological findings. (a) The macroscopic image of the excision. Involvement of the submandibular gland with the submandibular lymph node. There is no continuity with buccal mucosal carcinoma. Submandibular gland (red), mandibular bone (yellow), and buccal mucosal carcinoma (green). (b) The microscopic image of buccal mucosal carcinoma (HE staining: 9 × 20). Invasiveness of well-differentiated squamous cell carcinoma is observed. (c) The microscopic image of submandibular gland invaded with ENE of metastatic submandibular LN (HE staining: 20 × 20 and 20 × 100). Submandibular gland tissue above the dotted line and well-differentiated squamous cell carcinoma below the dotted line are seen.

Histopathological findings revealed the presence of multilobed tumor cells with pale acidophilic spores that formed small and large foci and proliferated in sheets. Extensive keratinized nests were observed in the center of the tumor foci. The nuclei of the tumor cells were round and enlarged, with irregular sizes, malformed nuclear borders, and clear nucleoli. The resection margins were negative with horizontal 3 mm and vertical 2.5 mm, and the tumor was well-differentiated, classified as Yamamoto-Kohama (YK) 3 with venous and perineural invasion. Four of the 70 LNs, including three SMLNs and one superior internal deep cervical LN were positive, and one SMLN had ENE involving the SMG (Figure 5(a)–5(c)). As a result, the histopathological diagnosis was SCC of the buccal mucosa, G1 (pT4bN3bM0, Stage IVB).

## 3. Discussion

The incidence of histopathological involvement of the SMG in OSCC is generally low, ranging from 0% to 5%. In 64.2% of cases, SMG contributes to OSCC through direct invasion from the primary carcinoma, while in 23.9% of cases, it contributes through direct ENE from SMLNs [1–3]. Our case of SMG involvement occurred due to direct ENE from SMLNs, which is a relatively rare occurrence.

Jakhetiya et al. reported that in 303 patients with OSCC who underwent ND, SMG involvement was found in four cases (1.32%), including one case of direct invasion from the primary tumor and three cases of metastasis from LN, and reported that the spread of perineural and ENE was associated with SMG involvement [4]. In their study of 330 patients with OSCC, Zeng et al. found SMG invasion in only seven cases (2.1%). Of these, five cases were direct invasion and two cases were ENE of SMLNs, all with T4 primary tumor and N2b or higher LN metastasis. In contrast, hematogenous and lymphogenous SMG metastases are extremely rare, and SMG preservation is considered safe in the absence of direct infiltration [5]. Spiegel et al. reported that 5.3% of the 169 patients had SMG involvement, three patients had direct invasion from SMLNs, six patients had direct invasion from the primary tumor, and there were no intraglandular LNs in the SMG [6]. Direct invasion is the primary pathogenesis of SMG invasion in OSCC, and hematogenous and lymphogenous metastasis are very rare. Previous reports of SMG involvement summarized the literature from 2004 to 2023, 67 SMG involvement of the 3,489 SMGs resected, 43 (64.2%) of 67 were directly invaded from the primary tumor, and 16 (23.9%) from ENE of SMLN metastasis [2]. The frequency of SMG invasion from SMLN metastasis was lower than that from the primary tumor. To the best of our knowledges, seven previously reported literatures on SMG involvement with ENE from SMLN were summarized. From 2004 to 2023, only 17 cases (0.97%) of 1,944 resected SMGs and 50 cases of SMG involvement were reported (Table 1) [2, 4–9].

On the contrary, it has been reported that 0.1% of cases involve the SMG from intraglandular LNs or Wharton's duct other than direct SMG involvement [10]. Involvement of the SMG from Wharton's duct is extremely rare and has only been reported in one case by Fives et al. The case involved direct extension of a carcinoma of the floor of the mouth through Wharton's duct into the SMGs on both sides, and histopathological examination confirmed the extension of SCC along Wharton's duct [11]. Basaran et al. reported intraglandular metastasis in only one out of 294 cases of tongue cancer but did not specify whether the metastasis was intraglandular or hematogenous [7]. DiNardo found that SMLNs around the SMG were divided into perivascular, anterior submandibular, posterior submandibular, deep, and intraglandular, but no intraglandular LNs were found [12]. Dhiwakar et al. reported that 30 SMG specimens excised by ND in 3 mm slices showed no intraglandular LNs or tumors [13]. Many studies have reported negative findings regarding the presence of intraglandular LNs.

Regarding the prognosis of SMG invasion related to OSCC, Chen et al. reported that all six cases were T4, N2b or higher, three cases died within 6 months due to local or regional recurrence, two cases survived for more than 3 years, and one case survived for more than 6 years [8]. The patient in the current case had multiple neck metastases and ENE invaded the SMG of pT4bN3bM0. Adjuvant therapy was not administered due to the patient's inability to take oral intake and the need for prolonged eating and swallowing rehabilitation, as well as advanced age. In our case, although the patient died from distant metastases, locoregional control was achieved by radical resection of the primary tumor and ND performed as one block with reconstructive surgery. Therefore, it was considered that treatment for radical therapy was better than palliative radiotherapy or BSC in case of SMG involvement.

## 4. Conclusion

SMG involvement from direct invasion from a metastatic SMLN is relatively rare. In our case, although the patient died from distant metastases, locoregional control was achieved through curative resection of the primary tumor and ND performed as one block with reconstructive surgery.

## Figures and Tables

**Table 1:** Reports of submandibular gland involvement from submandibular lymph node metastases.

**No.**	**Authors**	**Year**	**Total SMG**	**SMG involvement**	**(%)**	**Invasion from submandibular LN**	**(%)**
1	Spiegel JH	2004	196	9	4.59	3	1.53
2	Chen TC	2009	383	7	1.83	1	0.26
3	Basaran B	2013	294	13	4.42	4	1.36
4	Panda NK	2015	163	6	3.68	1	0.61
5	Zeng W	2019	363	7	1.93	2	0.55
6	Jakhetiya A	2021	366	4	1.09	3	0.82
7	Yamagata K	2023	179	4	2.23	3	1.68
Total			1944	50	2.83	17	0.97

Abbreviations: LN: lymph node, SMG: submandibular gland.

## Data Availability

The data supporting this study's findings are available from the corresponding author upon reasonable request.
